# TET enzymes control antibody production and shape the mutational landscape in germinal centre B cells

**DOI:** 10.1111/febs.14934

**Published:** 2019-06-03

**Authors:** Katia Schoeler, Andreas Aufschnaiter, Simon Messner, Emmanuel Derudder, Sebastian Herzog, Andreas Villunger, Klaus Rajewsky, Verena Labi

**Affiliations:** ^1^ Division of Developmental Immunology Biocenter, Medical University of Innsbruck Austria; ^2^ Institute for Biomedical Aging Research University of Innsbruck Austria; ^3^ CeMM Research Center for Molecular Medicine of the Austrian Academy of Sciences Vienna Austria; ^4^ Ludwig Boltzmann Institute for Rare and Undiagnosed Diseases Vienna Austria; ^5^ Max Delbrück Center for Molecular Medicine in the Helmholtz Association Berlin‐Buch Germany

**Keywords:** B cells, germinal centre, somatic hypermutation, TET2, TET3

## Abstract

Upon activation by antigen, B cells form germinal centres where they clonally expand and introduce affinity‐enhancing mutations into their B‐cell receptor genes. Somatic mutagenesis and class switch recombination (CSR) in germinal centre B cells are initiated by the activation‐induced cytidine deaminase (AID). Upon germinal centre exit, B cells differentiate into antibody‐secreting plasma cells. Germinal centre maintenance and terminal fate choice require transcriptional reprogramming that associates with a substantial reconfiguration of DNA methylation patterns. Here we examine the role of ten‐eleven‐translocation (TET) proteins, enzymes that facilitate DNA demethylation and promote a permissive chromatin state by oxidizing 5‐methylcytosine, in antibody‐mediated immunity. Using a conditional gene ablation strategy, we show that TET2 and TET3 guide the transition of germinal centre B cells to antibody‐secreting plasma cells. Optimal AID expression requires TET function, and TET2 and TET3 double‐deficient germinal centre B cells show defects in CSR. However, TET2/TET3 double‐deficiency does not prevent the generation and selection of high‐affinity germinal centre B cells. Rather, combined TET2 and TET3 loss‐of‐function in germinal centre B cells favours C‐to‐T and G‐to‐A transition mutagenesis, a finding that may be of significance for understanding the aetiology of B‐cell lymphomas evolving in conditions of reduced TET function.

## Graphical abstract

TET (ten‐eleven‐translocation) enzymes promote gene expression by catalyzing the oxidation of 5‐methylcytosine in DNA. TET deficiency has been linked to defects in embryonic development and enhanced risk of myeloid malignancies, including B‐cell lymphomas. Here, Verena Labi and colleagues show using a conditional gene ablation strategy that TET function is vital for humoral immunity *in vivo*. TET activity guides the transition of germinal center B cells to antibody‐secreting plasma cells and promotes antibody isotype switching (class‐switch recombination). Loss of TET function favors C‐to‐T and G‐to‐A mutagenesis during somatic hypermutation, a finding of potential significance for understanding the etiology of B‐cell lymphomas.

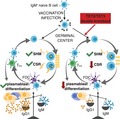

**Abbreviations**
5hmC: 5‐hydroxymethylcytosine
5mC: 5‐methylcytosine
AID: activation‐induced cytidine deaminase
BCR: B‐cell antigen receptor
CB: centroblast
CC: centrocyte
CGG: chicken gammaglobulin
CSR: class switch recombination
DLBCL: Diffuse Large B‐cell Lymphoma
DNMT: DNA methyltransferase
DZ: dark zone
FO: follicular
GC: germinal centre
iGC: induced germinal centre
LZ: light zone
MZ: marginal zone
NP: 4‐hydroxy‐3‐nitrophenylacetyl
PC: plasma cell
RBC: red blood cell
SHM: somatic hypermutation
TET: ten‐eleven‐translocation

## Introduction

Epigenetic regulation at the level of DNA is largely mediated by covalent addition of a methyl moiety at the 5th carbon of cytosines via DNA methyltransferases (DNMT) 1. 5‐methylcytosine (5mC), enriched in the context of CpG dinucleotides, associates with a local repression of gene expression 2. Embryonic development is accompanied by a vast turnover of DNA methylation, guiding developmental transitions and ultimately stabilizing cell identity. It has been assumed that the DNA methylation patterns that are established during embryonic development are self‐perpetuating and not substantially altered in adult tissues. Yet, mounting evidence suggests that context‐dependent changes in DNA methylation may be functional during postnatal lineage priming, commitment and cell function 3, 4, 5, 6, 7, 8.

Ten‐eleven‐translocation (TET) proteins (TET1, TET2 and TET3) catalyse the iterative oxidation of the 5mC methyl moiety to 5‐hydroxymethylcytosine (5hmC), 5‐formylcytosine and 5‐carboxylcytosine 9, 10, 11, 12, with 5hmC being 10‐ to 100‐fold more prevalent than its higher oxidized states 13. 5hmC is enriched in the gene bodies of highly transcribed genes and at active enhancers 14, 15, and 5hmC accumulation in the latter positively correlates with chromatin accessibility. Despite representing persistent epigenetic marks on their own, oxidized cytosine species are also functional intermediates for TET‐mediated DNA demethylation. Yet the underlying molecular mechanisms are poorly understood. Oxidized cytosines may facilitate replication‐dependent passive dilution of methylated cytosines, or mark 5mC for active replacement by unmodified cytosines through deamination and glycosylase‐dependent excision and repair 10, 12, 16, 17, 18.

*In vivo*, TET‐deficiency associates with the deregulation of embryonic development 19, and TET2 loss‐of‐function poses a risk for transformation such as in myeloid malignancies 20, 21, 22 or Diffuse Large B‐cell Lymphoma (DLBCL) 23, 24, the latter tumour originating from germinal centre (GC) B cells.

Germinal centres are transient structures in secondary lymphoid tissues that are initiated by B cells upon engagement of their cell surface B‐cell antigen receptor (BCR) by antigen, such as during pathogen infection or vaccination 25, 26. During the GC reaction, the enzyme activation‐induced cytidine deaminase (AID) is vital for the induction of somatic hypermutation (SHM) and class‐switch recombination (CSR) at the BCR gene loci. Clonally expanding GC B cells successively reprogramme their transcriptome, eventually differentiating into memory B cells or antibody‐secreting cells, that is plasma cells that produce large amounts of high‐affinity antibodies.

The transcriptional signatures that separate B‐cell progenitors from mature naïve B cells, and mature naïve B cells from functionally distinct progeny are established by ordered and stepwise reconfiguration of the DNA methylome 27, 28, 29, 30, 31. In humans, an estimated 30% of the entire DNA methylome is modified during B‐cell development, affecting several million CpG sites 30, 32. B‐cell‐specific deletion of DNMT1 in mice resulted in a complete block in early B‐cell development 33. Using conditional mouse models, we and others have demonstrated that a combined lack of TET2 and TET3 from early B‐cell development on prevents focal DNA hypomethylation at enhancers that are enriched for consensus binding motifs of key B‐lineage transcription factors, and consequently corrupts the gene expression programme associated with maturation transitions 34, 35. The transcription factors PU.1 and EBF1 were shown to interact with and possibly recruit TET2 to target regulatory elements 35, 36, suggesting a sequence‐specific mechanism of TET‐mediated DNA demethylation. Double‐deficiency of TET2 and TET3 partially blocked pro‐B to pre‐B‐cell transition, impaired BCR light chain rearrangement and resulted in reduced numbers of peripheral mature B cells. Of note, these mature B cells were nonresponsive to T‐cell‐dependent immunization 34.

Preventing DNA methylation has been shown to terminate GCs 33. In contrast, much remains to be learned about the role of TET enzymes in the GC response and for humoral immunity. To this aim, we generated a mouse model of acute joint ablation of TET2 and TET3 in established GC B cells, and report a vital role for TET function in antibody‐mediated immunity.

## Results and Discussion

### TET2 and TET3 are expressed throughout B‐cell development and terminal differentiation

Human and mouse B cells express TET2 and TET3, whereas TET1 is barely detectable throughout development and upon activation 37, 38, 39, 40. To analyse the relative mRNA expression of TET2 and TET3 in cell populations spanning the entire B‐cell lineage, we FACS‐sorted developing and mature naïve B‐cell subsets from unchallenged wild‐type mice and antigen‐experienced B cells from mice that were immunized with sheep red blood cells (RBC), and performed quantitative real‐time PCR (qRT‐PCR).

**Figure 1:**
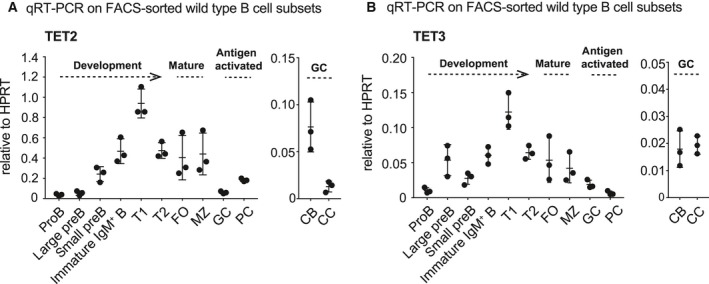
mRNA expression of TET2 and TET3 in B cells *ex vivo*. B‐cell populations were FACS‐sorted from bone marrow and spleens of 8‐wk‐young wild type mice (Development and Mature; *n* = 3) or spleens from 9‐wk‐young wild type mice that has been immunized with sheep RBC 7 days before (Antigen activated; *n* = 3), and RNA was isolated for qRT‐PCR analysis for (A) TET2 and (B) TET3 expression. HPRT was used as reference gene. Bone marrow: pro B cells, large and small pre B cells and immature IgM^+^ B cells. Spleen: transitional 1 (T1) B cells, transitional 2 (T2) B cells, mature follicular (FO) B cells, marginal zone (MZ) B cells, germinal centre (GC) B cells and plasmablasts/plasma cells (PC). GC B cells were further divided into dark zone centroblasts (CB) and light zone centrocytes (CC). Data are shown as mean ± SD.

During B‐cell development expression of both, TET2 (Fig. 1A) and TET3 (Fig. 1B) is progressively increasing, peaking in splenic transitional 1 B cells. Such a pattern indicates that the two proteins may have partially overlapping functions in developmental transitions or the selection of immature B cells, potentially controlling the appearance of autoimmune or nonfunctional mature B cells. However, a robust understanding of TET‐function in peripheral immature and mature naïve B‐cell subsets could only be achieved using conditional *Cre* transgenes with selective activity in the cell type of interest. As compared to mature naïve follicular (FO) B cells, TET2 and TET3 are substantially down‐regulated in antigen‐experienced GC B cells and plasma cells, a result in agreement with a recent report in human GC B cells 37 (compare Fig. 1A and B; FO vs. GC vs. PC). GC B cells cyclically migrate between the GC dark zone (DZ), where they undergo clonal expansion and SHM, and the GC light zone (LZ) where cells expressing a high‐affinity BCR are positively selected. Whereas TET3 mRNA is not differentially expressed between the DZ centroblasts (CB) and the LZ centrocytes (CC), TET2 reaches its lowest level in centrocytes. Altogether, these results indicate that TET2 and TET3 might serve both, overlapping and unique functions in antibody‐mediated immunity.

Dependent on the mouse strain and animal facility, young mice lacking only TET2 or TET3 display no or only moderate B‐cell phenotypes at steady state 34, 41, 42. Hence, we reasoned that only combined deletion of TET2 and TET3 may unravel significant TET‐dependent effects in B cells upon activation.

### Combined loss of TET2 and TET3 impairs plasmablast differentiation *in vitro*

B‐cell transit through the GC is accompanied by extensive DNA demethylation, focal methylation gains and an overall increased heterogeneity in DNA methylation patterns 30, 31, 43. DNMT1 hypomorphic mice present with a diminished GC size and abundance upon challenge 28. This suggests that maintenance of a specific DNA methylation pattern during the GC reaction is critical. Enforced DNA demethylation upon *in vitro* treatment of activated B cells with 5‐azacytidine augmented the appearance of plasmablasts in a division‐dependent manner 31. Conversely, inhibition of DNA demethylation might impair plasma cell generation.

Addressing the involvement of TET proteins in this process, we generated Cg1‐Cre*;Tet2* ^*F/F*^ *;Tet3* ^*F/F*^ mice in which physiologic germ‐line Cg1 transcription drives expression of the Cre‐recombinase 44. Using this system, joint Cre‐mediated deletion of both *Tet* genes is expected in a majority of GC B cells upon IgG1‐priming. Of note, acute GC B cell‐specific *Tet* deletion circumvents indirect effects caused by extended TET‐deficiency during B‐cell development.

First, we used a co‐culture system that allows the generation and exponential growth of *in vitro* induced GC (iGC) B cells 45. In this system, mature naïve B cells are cultured on feeder cells that stably express CD40 ligand and secrete BAFF thus mimicking T cell help. Dependent on the cytokine provided, that is exclusive exposure to IL‐4 for 8 days or initial exposure for 4 days to IL‐4 followed by IL‐21 for another 4 days, this culture allowed us to determine the dependency of iGC B cells on TET‐proteins for proliferation, CSR and plasmablast generation.

**Figure 2:**
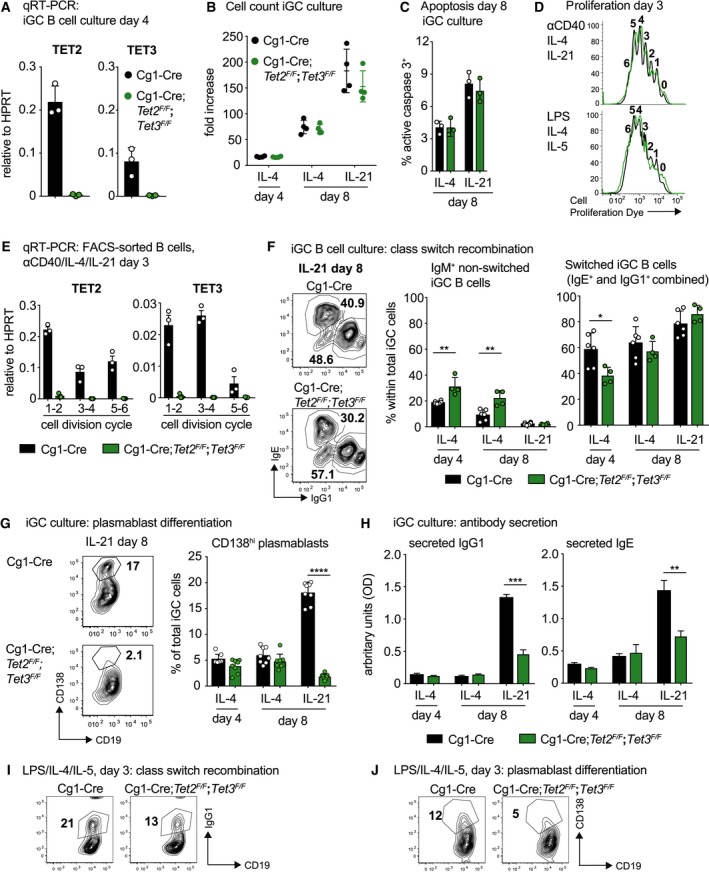
Combined loss of TET2 and TET3 impairs plasmablast differentiation *in vitro*. (A) qRT‐PCR analysis for TET2 and TET3 mRNA expression in iGC B cells cultivated *in vitro* for 4 days (*n* = 3 for each genotype). (B) Population doublings of iGC B cells cultured on 40LB feeder cells with IL‐4 for 4 days, and additional 4 days with either IL‐4 or IL‐21. Numbers of live cells were determined via Trypan blue exclusion (*n* = 4/genotype). (C) Intracellular flow cytometry analysis for active (cleaved) caspase 3 performed on iGC B cells cultivated *in vitro* for 8 days (*n* = 3/genotype/condition). (D) Splenic naïve B cells were labelled with a proliferation‐tracking dye (Cell Proliferation Dye eF450) and stimulated with αCD40/IL‐4/IL‐21 or LPS/IL‐4/IL‐5 for 72 h. Loss of the tracking dye was assessed by flow cytometry. Numbers above individual peaks represent the respective division‐cycles. Overlay histograms are representative of three mice per genotype. (E) qRT‐PCR analysis for TET2 and TET3 on αCD40/IL‐4/IL‐21‐stimulated FACS‐sorted B cells that had divided for a defined number of times (*n* = 3 for each genotype); cells were derived from the cultures described in (D) and for each biological replicate divisions 1–2, 3–4 and 5–6 were pooled into one sample. (F) Representative flow cytometry plots (left; day 8 with IL‐21) and bar graphs (right) depicting IgM^+^ or switched (IgG1^+^ and IgE^+^ combined) cells within total iGC B cells (*n* = 6 for Cg1‐Cre and *n* = 4 for Cg1‐Cre;*Tet2* ^*F/F*^;*Tet3* ^*F/F*^). Of note, the fraction of B cells neither IgM^+^, IgE^+^ nor IgG1^+^ (approximately 20% per sample) was not further investigated. (G) Representative flow cytometry plots (left; day 8 with IL‐21) and bar graphs (right) depicting the fraction of CD138^hi^ plasmablasts within total iGC B cells (*n* = 8 for Cg1‐Cre and *n* = 8 for Cg1‐Cre;*Tet2* ^*F/F*^;*Tet3* ^*F/F*^). (H) Bar graphs depicting ELISA results for antibodies secreted into the culture medium (*n* = 3 for Cg1‐Cre and *n* = 3 for Cg1‐Cre;*Tet2* ^*F/F*^;*Tet3* ^*F/F*^). (I) Flow cytometry plots depicting switched IgG1^+^ B cells upon stimulation with LPS/IL‐4/IL‐5 for 3 days. Plots are representative of three mice per genotype. (J) Flow cytometry plots depicting CD138^+^ plasmablasts upon stimulation with LPS/IL‐4/IL‐5 for 3 days. Plots are representative of three mice per genotype. Data are shown as mean ± SD. *P < 0.05, **P < 0.01, ***P < 0.005, ****P < 0.0001 vs Cg1‐Cre control (unpaired Student's *t*‐test).

After 4 days of iGC culture, acute *Tet* deletion is complete as indicated by qRT‐PCR analysis (Fig. 2A). Within the limited duration of the 8 days culture system, double‐deficiency of TET2 and TET3 did not alter cell growth, as indicated by an identical increase in cellularity between control and Cg1‐Cre*;Tet2* ^*F/F*^ *;Tet3* ^*F/F*^ iGC B cell cultures (Fig. 2B). This is consistent with a comparable fraction of apoptotic cells (Fig. 2C). To confirm in an independent culture system that TET‐deficiency does not impact the proliferation of activated B cells, naïve B cells were labelled with a proliferation‐tracking dye and stimulated with αCD40/IL‐4/IL‐21 or LPS/IL‐4/IL‐5. No alterations in proliferation between the genotypes were observed (Fig. 2D) despite the highly efficient and division‐independent deletion of *Tet2* and *Tet3* after 3 days in culture (Fig. 2E). In TET‐proficient B cells, both TET mRNAs were down‐regulated in a cell division cycle‐dependent manner, albeit with different kinetics. Whereas TET2 was initially down‐regulated and moderately up‐regulated in division cycles 5–6, down‐regulation of TET3 was only apparent once the cells had divided > 4 times. From these results a picture emerges where GC B cells down‐regulate TET proteins to prevent premature terminal differentiation, and up‐regulation of TET2 is required for optimal plasmablast differentiation. This is in line with Dominguez *et al*. 46 who propose that low TET2 levels, such as in patients with clonal hematopoiesis of indeterminate potential that present with TET2 loss‐of‐function mutations 47, prevent terminal differentiation hence facilitating B lymphomagenesis.

The cytokine milieu in the iGC culture system favours CSR from IgM to IgG1 or IgE. CSR to these two isotypes was significantly though moderately impaired in Cg1‐Cre*;Tet2* ^*F/F*^ *;Tet3* ^*F/F*^ cells (Fig. 2F). Strikingly, IL‐21‐driven differentiation into CD138^+^ plasmablasts, antibody‐secreting precursors of long‐lived plasma cells, was strongly diminished (Fig. 2G). Accordingly, the amount of IgG1 and IgE secreted into the medium was significantly reduced in TET2/TET3 double‐deficient iGC B cell cultures (Fig. 2H). The dependence of B cells on TET activity for CSR to IgG1 and plasmablast differentiation could be recapitulated using an independent culture system (Fig. 2I,J). Hence, our data suggest that TET function is essential for proper plasmacytic differentiation. TET2 might serve a dominant role, as it was shown to cause the demethylation of intronic CpGs in the *Prdm1* locus encoding BLIMP‐1 46, a key transcriptional repressor for plasmacytic differentiation.

We note that we have not yet assessed a causal relationship between TET activity and the methylation changes during plasmacytic differentiation. While the distribution of 5hmC has been determined in various B‐cell subsets 30, 31, technical limitations prevent so far a direct identification of the genomic regions where TET2 and TET3 exert their functions.

Targeted hypomethylation at *cis*‐regulatory sites might be initiated by the binding of transcription factors such as EBF1 35, that subsequently recruit TET proteins allowing for 5hmC deposition. Focally demethylated regions were found enriched for binding motifs for NF‐κB and AP‐1 or IRF4 and Oct‐2 transcription factors during early and late phases of plasmablast differentiation respectively 31. However, how transcription factor‐assisted recruitment of TET enzymes and consequential oxidation of 5mC result in targeted DNA demethylation is still a matter of debate. 5hmC‐mediated DNA demethylation may occur in an enzyme‐dependent active, or a replication‐coupled passive manner 40. Given that plasmablast differentiation is tightly coupled to cell division 43, it is likely that passive dilution of oxidized cytosine species accounts for a substantial fraction of the observed global demethylation. In accordance, a recent report has described a progressive replication‐dependent loss of 5hmC in activated B cells in the absence of TET function 40. Nevertheless, as TET2/TET3 double‐deficiency does not impair cell growth in culture (Fig. 2B,D) despite preventing plasmacytic differentiation (Fig. 2G,H,J), our results support the idea that TET function during plasmablast generation does not depend on proliferation. This is consistent with a report by Caron *et al*. 43 concluding that DNA demethylation during plasmacytic differentiation *in vitro* is associated with the deposition of 5hmC at regions undergoing targeted demethylation, independent of proliferation. Next, we turned our attention to the humoral immune response *in vivo*.

### TET‐deficiency restricts IgG1 production *in vivo*

**Figure 3:**
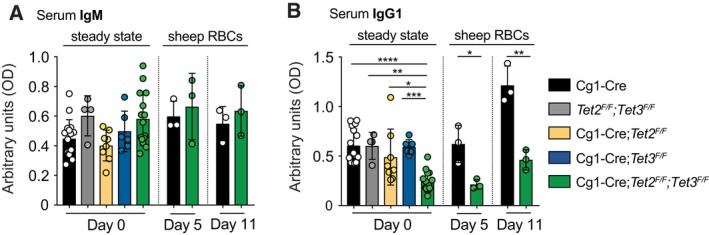
TET‐deficiency restricts the levels of circulating IgG1 *in vivo*. ELISA for IgM (A) and IgG1 (B) was performed on sera from unchallenged (steady state; *n* = 16 for Cg1‐Cre, *n* = 4 for *Tet2* ^*F/F*^;*Tet3* ^*F/F*^, *n* = 8 for Cg1‐Cre;*Tet2* ^*F/F*^, *n* = 6 for Cg1‐Cre; *Tet3* ^*F/F*^ and *n* = 15 for Cg1‐Cre;*Tet2* ^*F/F*^;*Tet3* ^*F/F*^ mice) and sheep RBC‐immunized mice (days 5 and 11; *n* = 3 for Cg1‐Cre and *n* = 3 for Cg1‐Cre;*Tet2* ^*F/F*^;*Tet3* ^*F/F*^). Data are shown as mean ± SD. *P < 0.05, **P < 0.01, ***P < 0.005, ****P < 0.0001 vs Cg1‐Cre;*Tet2* ^*F/F*^;*Tet3* ^*F/F*^ mice (One‐way ANOVA and Tukey *post‐hoc* test for multiple comparisons; unpaired Student's *t*‐test when comparing two groups).

We assessed the levels of antibodies circulating in the peripheral blood of TET2/TET3 double‐deficient mice at steady‐state. Of note, Cg1‐Cre‐mediated recombination mostly involves IgG1, whereas IgM^+^ B cells largely retain the *loxP*‐flanked gene 44. In accordance, steady‐state levels of IgM in peripheral blood were unchanged in Cg1‐Cre*;Tet2* ^*F/F*^ *;Tet3* ^*F/F*^ mice (Fig. 3A). However, TET activity was required for optimal IgG1 titres, as indicated by significantly reduced basal IgG1 levels (Fig. 3B). Of note, neither TET2‐ nor TET3‐deficiency resulted in alterations in steady‐state IgG1 levels, indicating compensatory potential during steady‐state humoral immunity (Fig. 3B).

To determine whether GC B cell‐specific TET2/TET3 double‐deficiency manifests defects in the magnitude of antibody production *in vivo*, we induced T‐cell‐dependent humoral immunity by challenging Cg1‐Cre control and Cg1‐Cre*;Tet2* ^*F/F*^ *;Tet3* ^*F/F*^ mice with sheep RBC. GC B cells that form in response to RBC antigens predominately switch to IgG1. Peripheral blood was collected at days 5 and 11 post immunization, and ELISA was performed to determine the abundance of serum antibody isotypes. Whereas IgG1 titers increased upon immunization in Cg1‐Cre mice, Cg1‐Cre*;Tet2* ^*F/F*^ *;Tet3* ^*F/F*^ mice displayed only a marginal increase in circulating IgG1 from day 0 to day 11 (Fig. 3B).

These *in vivo* data mirror the defects in antibody production observed *in vitro* (Fig. 2H), and indicate that complete TET loss‐of‐function impairs humoral immunity in a GC B‐cell‐autonomous manner.

### Loss of TET‐function impairs GC maintenance and CSR upon immunization

**Figure 4:**
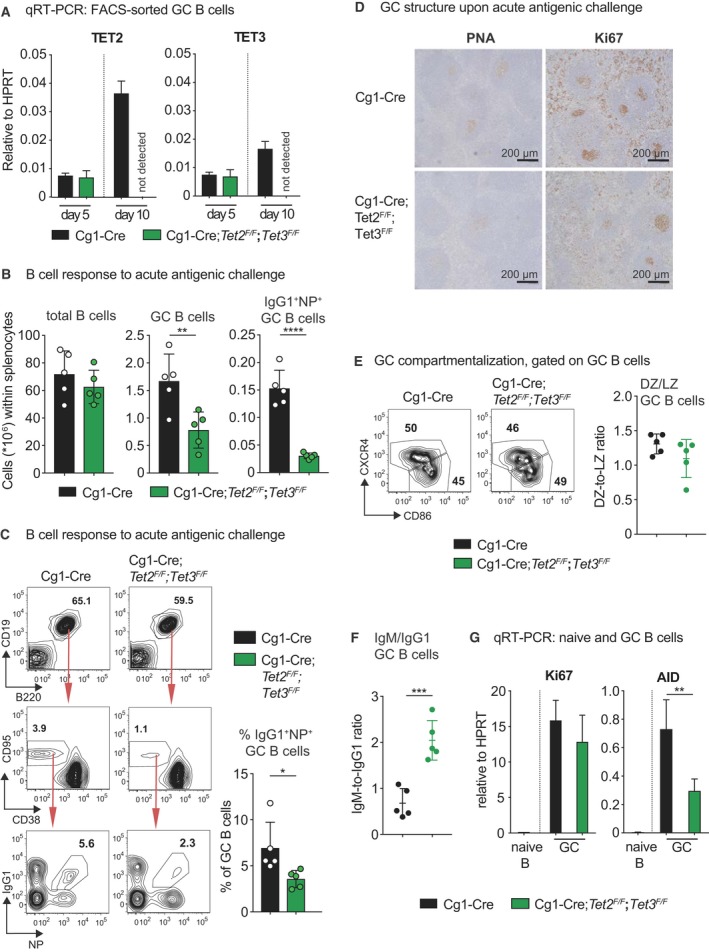
Loss of TET‐function impairs GC maintenance and CSR upon immunization. (A) qRT‐PCR analysis for TET2 and TET3 mRNA expression in FACS‐sorted splenic GC B cells of the indicated genotypes 5 and 10 days post immunization with NP‐CGG (*n* = 3 for Cg1‐Cre and *n* = 3 for Cg1‐Cre;*Tet2* ^*F/F*^;*Tet3* ^*F/F*^). (B) Mice of the indicated genotypes were immunized at the age of 8–10 weeks with NP‐CGG and analysed 14 days later. Flow cytometry analysis of splenic single‐cell suspensions identified the fractions of total B cells (B220^+^ CD19^+^), GC B cells (CD19^+^B220^+^ CD138^−^ CD95^hi^ CD38^lo/−^) and NP ^+^IgG1^+^ GC B cells within total splenocytes. Total cell numbers for the respective cell populations are shown (*n* = 5 for Cg1‐Cre and *n* = 5 for Cg1‐Cre;*Tet2* ^*F/F*^;*Tet3* ^*F/F*^). (C) Representative flow cytometry plots (left) and bar graph (right; *n* = 5 for Cg1‐Cre and *n* = 5 for Cg1‐Cre;*Tet2* ^*F/F*^;*Tet3* ^*F/F*^) depict fractions of B cells, GC B cells and IgG1^+^ NP ^+^ GC B cells shown in (B). Arrows indicate gating strategy. (D) Spleen sections from the indicated genotypes 14 days post immunization with NP‐CGG were analysed for Ki67 (proliferating cells) and for the ability of cells to bind PNA (GC B cells). The brown precipitate indicates positive staining. Sections were counterstained with haematoxylin. One representative mouse out of three per group is shown. Scale bars: 200 μm. (E) Representative flow cytometry plots (left) depict fractions of DZ and LZ GC B cells within total GC B cells. Scatter graph (right; *n* = 5 for Cg1‐Cre and *n* = 5 for Cg1‐Cre;*Tet2* ^*F/F*^;*Tet3* ^*F/F*^) shows the ratio of DZ and LZ GC B cells. (F) Scatter graph depicts the ratio of IgM^+^ and IgG1^+^ cells within GC B cells (*n* = 5 for Cg1‐Cre and *n* = 5 for Cg1‐Cre;*Tet2* ^*F/F*^;*Tet3* ^*F/F*^). (G) qRT‐PCR analysis for Ki67 and AID mRNA in FACS‐sorted naïve Cg1‐cre B cells (*n* = 3) and GC splenic B cells (*n* = 3 for Cg1‐Cre and *n* = 3 for Cg1‐Cre;*Tet2* ^*F/F*^;*Tet3* ^*F/F*^) 14 days post NP‐CGG immunization. Data are shown as mean ± SD. *P < 0.05, **P < 0.01, ***P < 0.005, ****P < 0.0001 vs Cg1‐Cre control (Student's *t*‐test).

To explore the role of TET proteins in the GC response, we used an alum‐adsorbed hapten‐carrier conjugate, 4‐hydroxy‐3‐nitrophenylacetyl (NP) conjugated to chicken gamma globulin (NP‐CGG). NP‐specific GC B cells that form in response to alum‐adjuvanted immunogens predominately switch to IgG1 48, 49. Joint *Tet2* and *Tet3* deletion in Cg1‐Cre*;Tet2* ^*F/F*^ *;Tet3* ^*F/F*^ GC B cells was efficient by day 10 post immunization (Fig. 4A). Fourteen days post immunization, flow cytometry analysis revealed a substantially decreased overall and NP‐specific GC response in Cg1‐Cre*;Tet2* ^*F/F*^ *;Tet3* ^*F/F*^ mice, whereas the number and fraction of naïve B cells remained unaltered (Fig. 4B,C). Of note, within the fraction of GC B cells NP‐specific IgG1^+^ B cells were further reduced in Cg1‐Cre*;Tet2* ^*F/F*^ *;Tet3* ^*F/F*^ mice (Fig. 4C, bar graph). A reduced number of GC was also evident when performing immunohistochemistry for PNA and Ki67 on splenic sections of immunized mice (Fig. 4D). Despite the differential expression of TET2 between dark and light zone GC B cells (Fig. 1A), compartmentalization was not phenotypically altered in Cγ1‐cre*;Tet2* ^*F/F*^ *;Tet3* ^*F/F*^ mice (Fig. 4E). Within the total GC B‐cell population we observed a striking deficit for CSR from IgM to IgG1 (Fig. 4F), in accordance with the impaired CSR observed *in vitro* (Fig. 2F). Overall, these results indicate that TET proteins positively control the magnitude of the GC reaction, and facilitate CSR to IgG1.

Ki67 mRNA levels are unaltered in FACS‐sorted Cg1‐Cre*;Tet2* ^*F/F*^ *;Tet3* ^*F/F*^ GC B cells, suggesting that these cells proliferate normally (Fig. 2G). A recent report showed that the bZIP transcription factor BATF recruits TET proteins to TET‐responsive regulatory elements in the AID gene locus, promoting 5hmC deposition and demethylation, sustaining chromatin accessibility and bolstering AID expression 50. Consistent with this report and the observed CSR defect in Cg1‐Cre*;Tet2* ^*F/F*^ *;Tet3* ^*F/F*^ GC B cells, AID mRNA levels were found decreased by 50% in GC B cells (Fig. 4G) 50. Despite the fact that optimal AID expression is required for efficient CSR 50, transcriptional regulation of AID may not be the only mechanism by which TET proteins mediate AID function. Recently, AID‐mediated recruitment of TET2 to the *FANCA* locus and associated demethylation has been described in human lymphoma cell lines 39. Hence, it appears possible that AID recruits TET enzymes to further gene loci during somatic hypermutation. We wondered whether the reduced AID levels would impair affinity maturation in Cg1‐Cre*;Tet2* ^*F/F*^ *;Tet3* ^*F/F*^ GC B cells.

### Cg1‐Cre*;Tet2* ^*F/F*^ *;Tet3* ^*F/F*^ mice show compromised serum α‐NP IgG1 levels

**Figure 5:**
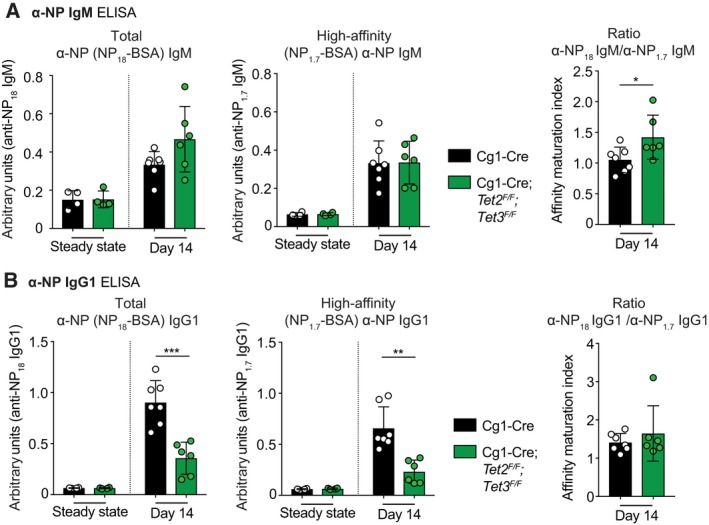
Cg1‐Cre*;Tet2* ^*F/F*^ *;Tet3* ^*F/F*^ mice show compromised serum α‐NP IgG1 levels. (A) Serum total (α‐NP_18_‐BSA) and high‐affinity (α‐NP_1.7_‐BSA) α‐NP antibody titres of the (A) IgM and (B) IgG1 isotypes before (steady state; *n* = 4 for Cg1‐Cre and *n* = 4 for Cg1‐Cre;*Tet2* ^*F/F*^;*Tet3* ^*F/F*^) or 14 days after (day 14; *n* = 7 for Cg1‐Cre and *n* = 6 for Cg1‐Cre;*Tet2* ^*F/F*^;*Tet3* ^*F/F*^) immunization with NP‐CGG. The maturation index is defined as the ratio of the α‐NP_18_ and α‐NP_1.7_ antibody titres. Data are shown as mean ± SD. *P < 0.05, **P < 0.01, ***P < 0.005 vs Cg1‐Cre control (Student's *t*‐test).

Following immunization with NP‐CGG, we determined the abundance and affinity of serum antibodies specific to NP by ELISA. Cg1‐Cre*;Tet2* ^*F/F*^ *;Tet3* ^*F/F*^ mice produced nearly similar amounts of NP‐specific IgM antibodies compared with Cg1‐Cre control mice at 14 days post immunization (Fig. 5A). In contrast, immunized Cg1‐Cre*;Tet2* ^*F/F*^ *;Tet3* ^*F/F*^ mice had substantially less circulating NP‐specific IgG1 in the peripheral blood as compared to Cg1‐Cre control mice (Fig. 5B). Despite a two‐fold reduction in NP‐specific IgG1, the levels of both total (NP_18_‐BSA) and high‐affinity (NP_1.7_‐BSA) α‐NP IgG1 were decreased similarly, resulting in no significant skewing of the affinity ratio in Cg1‐Cre*;Tet2* ^*F/F*^ *;Tet3* ^*F/F*^ mice (Fig. 5B).

Within the limits of this experimental approach, our results indicate that TET activity compromises α‐NP IgG1 quantity but not quality. The lack of phenotype in affinity maturation appeared surprising given the reduced levels of AID in Cg1‐Cre*;Tet2* ^*F/F*^ *;Tet3* ^*F/F*^ GC B cells (Fig. 4G), and motivated us to explore SHM in more detail.

### Loss of TET function does not impair affinity maturation but alters the mutation spectrum

Antibody affinity increases throughout affinity maturation, ultimately leading to the generation of highly protective neutralizing antibodies. The NP‐CGG model is amenable to SHM analysis as humoral immune responses against carrier‐coupled NP are dominated by B cells carrying the V_H_186.2 gene rearrangement 51, 52. We FACS‐sorted B220^+^CD19^+^CD138^−^CD95^hi^CD38^lo/−^IgG1^+^ cells from the spleens of three Cg1‐Cre control and three Cg1‐Cre*;Tet2* ^*F/F*^ *;Tet3* ^*F/F*^ mice 14 days post immunization. After RNA isolation, a region of 315 nucleotides of the V_H_186.2‐constant region g1 gene encompassing CDR1 and CDR2 was amplified, sequenced and mutations in 30 unique GC B‐cell clones per genotype were analysed by comparison with the germ‐line V_H_186.2 gene sequence.

**Figure 6:**
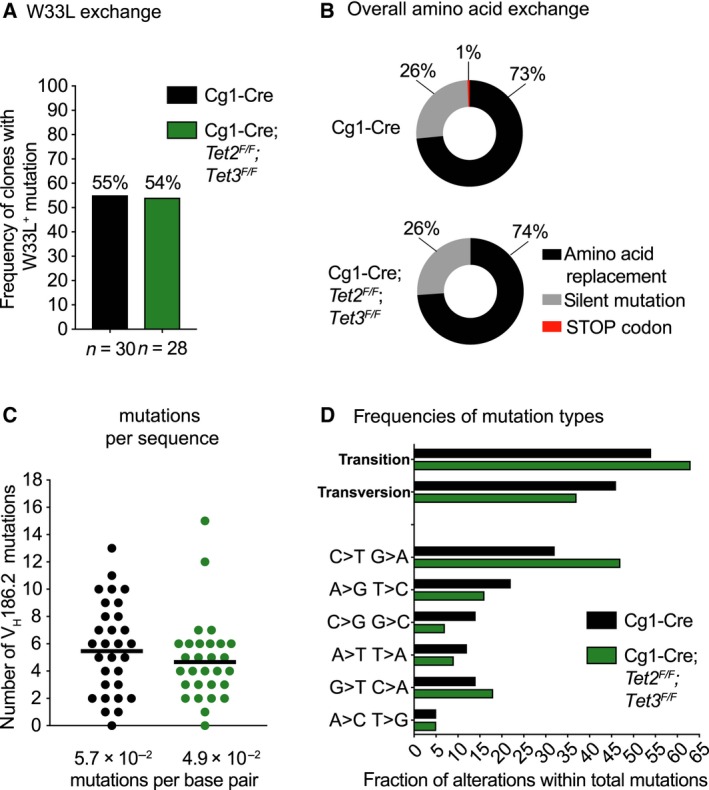
Loss of TET function does not prevent affinity maturation but alters the mutation spectrum. (A) IgG1^+^ GC B cells were FACS‐sorted from three Cg1‐Cre and three Cg1‐Cre;*Tet2* ^*F/F*^;*Tet3* ^*F/F*^ mice 14 days post immunization with NP‐CGG, and 10 unique V_H_186.2 γ1 transcripts per mouse were sequenced. Bar graph depicts the frequency of the W33L affinity‐enhancing mutation within the pool of unique GC B‐cell clones for each genotype. (B) Pie charts depict the % of V_H_186.2 mutations that are silent, lead to an amino acid replacement or a STOP codon. (C) Column scatter graph depicts the number of mutations in unique V_H_186.2 BCR sequences for each genotype. Data were analysed with Mann–Whitney with mean displayed. (D) Bar graph depicts the frequency of total and individual nucleotide transitions or transversions within the total number of mutations pooled for each genotype. *n* = 30 correct sequences for Cg1‐Cre and *n* = 28 correct sequences for Cg1‐Cre;*Tet2* ^*F/F*^;*Tet3* ^*F/F*^.

The well‐characterized tryptophan to leucine switch at position 33 of V_H_186.2 (W33L) 53, conferring a 10‐fold increase in the BCR affinity for NP, was comparably frequent in Cg1‐Cre control (55%) and Cg1‐Cre*;Tet2* ^*F/F*^ *;Tet3* ^*F/F*^ (54%) GC B‐cell clones (Fig. 6A). These data are in accordance with the ELISA results in Fig. 5. Furthermore, NP‐specific GC B cells from Cg1‐Cre*;Tet2* ^*F/F*^ *;Tet3* ^*F/F*^ mice exhibited similar fractions of amino acid replacement and silent mutations across the entire length of V_H_186.2 (Fig. 6B). Hence, SHM appears phenotypically unaltered in GC B cells deficient for TET2 and TET3.

Our current analysis is limited to the hapten NP, a simple antigen where a few specific mutations suffice for efficient affinity maturation. It will be interesting in the future to test the impact of TET activity on the formation of broadly neutralizing antibodies upon infection with pathogens carrying complex antigens, such as HIV or influenza that are characterized by extraordinary frequencies of affinity‐enhancing mutations.

Methylated CpG dinucleotides are a mutation hotspot in human cancers, a phenomenon that has been linked to the propensity of 5mC to deaminate. Hydroxymethylation of cytosines has been reported to enhance 54 or reduce 55 the likelihood of mutations. Hence, the modification state of cytosine likely influences the mutability of DNA, however, the relationship between mutability and cytosine modifications remains poorly understood. We found that Cg1‐Cre*;Tet2* ^*F/F*^ *;Tet3* ^*F/F*^ GC B cells carried slightly less somatic mutations per V_H_186.2 sequence (Fig. 6C), though this difference did not reach statistical significance. Strikingly, however, V_H_186.2 mutations in TET‐deficient GC B cells exhibited C‐to‐T and G‐to‐A transition mutation biases (Fig. 6D). In GC B cells, mutagenesis is largely a consequence of AID activity 56. In accordance with our results, B‐cell‐specific *Tet2*‐deficiency promoted age‐dependent transformation in a fraction of mice, an outcome that strictly required AID and involved a bias towards G‐to‐A and C‐to‐T mutations in the established tumours 38. This suggests that TET proteins may at least partially exert their tumour suppressor activity in GC B cell‐derived lymphomas by orchestrating the mutagenic potential of AID. One possible scenario is that cytidine deaminases (AID and APOBEC1‐3) have substantially lower activity on 5hmC compared to 5mC 57, 58. In addition, deamination of 5mC produces thymine, whereas 5hmC deaminates to 5‐hydroxymethyluracil, an atypical base that might be repaired more efficiently 55. Alternatively, 5hmC generated by TET enzymes may promote chromosome stability by supporting DNA damage repair 59. Up to 10% of human DLBCL exhibit *TET2* loss‐of‐function mutations, and these mutations usually occur mutually exclusive with mutations in regulators of TET function 46, 60. Finally, reduced TET function may actively impact on the aetiology of GC B‐cell‐derived tumours by shaping the mutational landscape or increasing chromosomal aberrations.

## Concluding remarks

The major goal of this study was to explore whether TET activity affects the magnitude or quality of humoral immune responses. Our data emphasize an essential role of TET function in GC expansion, plasmacytic differentiation and antibody production. The observed relative reduction in CSR correlated with decreased AID levels. In our model system, affinity maturation did not depend on TET function and was not limited by reduced AID levels. Strikingly, however, TET proteins appear to influence the mutagenic potential of AID as TET deficient GC B cells showed mutational skewing towards transition mutations, a function that may be of relevance for the aetiology of GC B‐cell‐derived lymphomas and potentially further tumour entities.

## Materials and methods

### Mice and immunization

The Cg1‐Cre 44, *Tet2* ^*F*^ 61 and *Tet3* ^*F*^ 42 alleles have been described. Male and female littermate mice were used indiscriminately in this study. Eight‐ to ten‐week‐old mice were immunized i.p. with 100 μg NP‐CGG/mouse in a volume of 200 μL per mouse. Alternatively, mice were injected i.p. with 1 × 10^9^ sheep RBCs (Labor Dr. Merck, Ochsenhausen, Germany, E‐400) in PBS in a volume of 200 μL per mouse. Animal procedures were approved by the Austrian Federal Ministry of Education, Science and Research (BMWF: 66‐011/0106‐WF/3b/2015 and 66‐011/0031‐V/3b/2019).

### Preparation of alum‐precipitated NP‐CGG

A freshly prepared 10% Alum solution [KAl(SO_4_)_2_ (Sigma, Saint Louis, MO, USA, 31242) in PBS] was mixed with a similar volume of 1 mg·mL^−1^ NP_15_‐CGG (Biosearch Technologies, Hoddeston, UK, N‐5055B‐5; prepared in PBS), and the pH was adjusted to 6.5–7.0 using 10 m NaOH (pH indicator; Macherey‐Nagel, Düren, Germany, 92118). The precipitate was washed three times with PBS at 2348 ***g*** for 15 s and finally resuspended in PBS to reach the initial volume of the Alum/NP‐CGG mixture.

### Preparation of primary single‐cell suspensions and cell counting

Staining buffer [PBS/3% FBS (Gibco, Grand Island, NY, USA, 10270‐106)] was generally used for cell preparation. Single‐cell suspensions were prepared by forcing murine spleen through 70 μm mesh filters (Corning, Cambridge, MA, USA, 352350), or flushing both femurs and tibiae using a 23G needle. Erythrocytes were depleted by incubating cells for 3 min in 1 mL lysis buffer (155 mm NH_4_Cl, 10 mm KHCO_3_, 0.1 mm EDTA; pH7.5) on ice. Cells were washed with staining buffer and filtered through a 50 μm cup filcon (BD Biosciences, San Diego, CA, USA, 340632), and cell numbers were determined using a hemocytometer and trypan blue exclusion.

### iGC B cell culture

B cells were enriched from splenic single‐cell suspensions using MagniSort Streptavidin Negative Selection Beads (Thermo Fisher Scientific, Waltham, MA, USA, MSNB‐6002‐74) as per manufacturer's instructions, and biotinylated antibodies against Ter119 (BioLegend, San Diego, CA, USA, 116204, 1 : 100), CD11b (BioLegend, 101204, 1 : 100) and TCRβ (BioLegend, 109204, 1 : 50).

The iGC B cell culture was conducted as described by Nojima *et al*. 45. Briefly, 40LB feeder cells were expanded in feeder cell medium: DMEM (Sigma, WHMISDZB) supplemented with 10% FBS (Gibco, 10270‐106), 2 mm l‐glutamine (Sigma, G7513) and 100 U·mL^−1^ penicillin/100 μg·mL^−1^ streptomycin (Sigma, 0781). 3 × 10^6^ feeder cells per 10 cm plate were treated for 2 h with 10 μg·mL^−1^ mitomycin C (Sigma, M0305) in 6 mL feeder cell medium. Following five repeated washing steps with PBS, 1.5 × 10^6^ B cells/10 cm dish were plated on 40LB feeder cells in 40 mL B‐cell medium: DMEM supplemented with 10% (v/v) FBS, 2 mm l‐glutamine, 10 mm Hepes (LONZA, Basel, Switzerland, BE17‐737E), 1 mm sodium pyruvate (Gibco, 13360‐039), 1× nonessential amino acids (Gibco, 11140‐035), 100 U·mL^−1^ penicillin/100 μg·mL^−1^ streptomycin, 50 μm β‐mercapto‐ethanol (Sigma, M3148) and 10 ng·mL^−1^ rIL‐4 (Peprotech, Rocky Hill, NJ, USA, 214‐14). On day 3, 30 mL of medium containing IL‐4 were replaced. On day 4, iGC B cells were harvested and analysed. 1.5 × 10^6^ iGC B cells were replated per 10 cm dish containing fresh mitomycin C‐treated 40LB feeder cells as detailed above, and cultivated in 40 mL of B‐cell medium containing either 10 ng·mL^−1^ rIL‐4 or 10 ng·mL^−1^ rIL‐21 (Peprotech, 210‐21). Thirty millilitre of medium containing the respective cytokines were replaced by fresh medium on day 7. The final analysis was conducted on day 8. Analyses (day 4 and 8) entailed collection of medium for ELISA and pellets for qRT‐PCR, cell counting (Trypan blue exclusion) and flow cytometric analysis. For analysis, iGC B cells were generally handled at room temperature and cultured at 37 °C in a humidified atmosphere containing 5% CO_2_.

### *In vitro* stimulation of B cells

Primary B cells were enriched from splenic single‐cell suspensions and cultivated in medium as described above for the iGC B‐cell culture at 37 °C in a humidified atmosphere containing 5% CO_2_. Mitogenic stimuli were used as follows: 1 μg·mL^−1^ α‐CD40 (Thermo Fisher Scientific, 16‐0402‐85), 25 ng·mL^−1^ IL‐4 (Peprotech, 214‐14), 10 ng·mL^−1^ IL‐21 (Peprotech 210‐21), 20 mg·mL^−1^ lipopolysaccharide (Sigma, L2880) and 25 ng·mL^−1^ IL‐5 (Peprotech, 215‐15).

To determine cell proliferation in the presence of mitogens *in vitro*, B cells were labelled with 10 μm Cell Proliferation Dye eFluor 450 (Thermo Fisher Scientific, 65‐0842‐90) as per manufacturer's instructions and placed in culture.

### Flow cytometry analysis of *ex vivo* isolated cells

Flow cytometry analysis has been performed as described in Ref. 62 using the following fluorochrome‐labelled antibodies: αB220‐PerCP/Cy5.5 (BioLegend, 103236, 1 : 300), αB220‐BV510 (BioLegend, 103247, 1 : 300), αCD19‐BV605 (BioLegend, 115540, 1 : 300), αCD19‐BV421 (BioLegend, 115538, 1 : 300), αAA4.1‐PE/Cy7 (BioLegend, 136507, 1 : 400), αAA4.1‐APC (BioLegend, 136510 1 : 400), αIgM F(ab′)_2_‐FITC (Jackson ImmunoResearch, 115‐096‐072, 1 : 300), αCD25‐PE (BioLegend, 102007, 1 : 400), αcKit‐APC (BioLegend, 135108, 1 : 300), αCD1d‐PE (Thermo Fisher Scientific, 12‐0011‐82, 1 : 500), αCD23‐PE/Cy7 (BioLegend, 101614, 1 : 400), αCD38‐FITC (BioLegend, 102705, 1 : 300), αCD38‐APC (BioLegend, 102712, 1 : 400), αCD38‐eFluor450 (Thermo Fisher Scientific, 48‐0381‐80, 1 : 300), αCD95‐PE (BD Biosciences, 554258, 1 : 300), αCD95‐PE/Cy7 (BD Biosciences, 557653, 1 : 800), αCXCR4‐APC (BioLegend, 146508, 1 : 100), αCD86‐PE/Cy7 (BioLegend, 105014, 1 : 100), αIgG1‐FITC (BD Biosciences, 553443, 1 : 200), αIgG1‐PE (BD Biosciences, 550083, 1 : 200), CD138‐PE (BioLegend, 142504, 1 : 300) and CD138‐BV510 (BioLegend, 142521, 1 : 200). NP_24_‐PE (Biosearch Technologies, N‐5070‐1, 1 : 500) was used to detect NP‐binding B cells. Data were acquired on an LSRII cytometer (BD Biosciences) and analysed using flowjo software (Tree Star, Ashland, OR, USA). Nonsinglet events were excluded from analyses using FSC‐H/FSC‐W and SSC‐H/SSC‐W characteristics.

### Flow cytometry analysis of iGC B cells

To collect iGC B cells from 10 cm dishes, 9/10 of the medium was gently removed, and the cells were incubated for 5 min at 37 °C in 3 mL harvest buffer (PBS/0.5% BSA/2 mm EDTA). Cells were harvested by addition of B‐cell medium and vigorous pipetting.

For CSR analysis, 5 × 10^5^ iGC B cells were washed twice with 1 mL PBS at room temperature and 460 ***g*** for 4 min. Subsequently, the cells were resuspended in 250 μL of 37 °C 0.05% trypsin (Sigma, T4174) and surface digested for 4 min at 37 °C. To stop trypsin digestion, 3 mL of staining buffer (described above for flow cytometry) were added, the cells were pelleted at 1800 r.p.m. for 4 min and the supernatant was removed. The cells were transferred to a U‐bottom 96 well plate and dead cells were labelled with the Fixable Viability Dye eFluor 780 (Thermo Fisher Scientific, 65‐0865‐14) as per manufacturer's instructions. Subsequently, cells were fixed and processed using the active Caspase 3 staining kit (BD Biosciences, 550914) as per manufacturer's instructions. Before staining with the active Caspase 3 antibody, the iGC B cells were incubated with 1 μg·mL^−1^ of αCD16/32 Fc‐Block (BioLegend, 101310) in 25 μL perm/wash buffer for 10 min at 4 °C. Subsequently another 25 μL of αIgE‐bio (BD Biosciences, 553419, 1 : 400) diluted in perm/wash buffer was added, the cells were further incubated for 15 min at 4 °C and washed with perm/wash buffer at 568 ***g*** for 2 min. Next, the cells were incubated with 25 μL of antibody mix containing αIgM‐PeCy7 (Thermo Fisher Scientific, 25‐5790‐81, 1 : 500), αIgG1‐FITC (BD Biosciences, 562026, 1 : 300), αCD19‐BV605 (BioLegend, 115540, 1 : 300) and Streptavidin‐APC (BioLegend, 405207, 1 : 800) for 15 min at 4 °C. The cells were washed with perm/wash buffer at 2000 r.p.m. for 2 min and stained for active caspase 3 as per manufacturer's instructions.

The processing of cells for plasmablast flow cytometry analysis was performed at room temperature in the dark. 5 × 10^5^ iGC B cells were transferred to a U‐bottom 96 well plate and pelleted at 2000 r.p.m. for 2 min. Dead cells were labelled with the Fixable Viability Dye eFluor 780 (Thermo Fisher Scientific, 65‐0865‐14) as per manufacturer's instructions. Subsequently, cells were incubated with 1 μg·mL^−1^ of αCD16/32 Fc‐Block in 25 μL staining buffer (PBS/3%FBS) for 10 min. Next, 25 μL of staining buffer containing αCD19‐BV605 (BioLegend, 115540, 1 : 300) and αCD138‐BV421 (BioLegend, 142508, 1 : 300) was added. The cells were further incubated for 15 min, washed with 200 μL of staining buffer at 2000 r.p.m. for 2 min, and resuspended in 200 μL staining buffer for flow cytometry analysis.

Data were acquired on an LSRII cytometer (BD Biosciences) and analysed using flowjo software (Tree Star). Nonsinglet events were excluded from analyses using FSC‐H/FSC‐W and SSC‐H/SSC‐W characteristics.

### Cell sorting

Cell sorting has been performed as described in Ref. 62. Briefly, bone marrow or splenic single‐cell suspensions were pre‐incubated with 1 μg·mL^−1^ of αCD16/32 Fc‐Block in 500 μL staining buffer (PBS/3%FBS) for 10 min, washed and stained for 20 min with antibodies in a volume of 500 μL staining buffer. The sorted cell subsets were defined as follows: Bone Marrow: pro B cells (B220^lo^CD19^+^AA4.1^+^IgM^−^CD25^−^ckit^+^), large pre B cells (B220^lo^CD19^+^AA4.1^+^IgM^−^CD25^+^ckit^−^FSC^hi^), small pre B cells (B220^lo^CD19^+^AA4.1^+^IgM^−^CD25^+^ckit^−^FSC^lo^) and immature IgM^+^ B cells (B220^lo^CD19^+^AA4.1^+^IgM^+^). Spleen: T1 B cells (CD19^+^B220^+^AA4.1^+^CD23^−^IgM^hi^), T2 B cells (CD19^+^B220^+^AA4.1^+^CD23^+^IgM^hi^), FO B cells (CD19^+^B220^+^AA4.1^−^CD1d^+^IgM^+^), MZ B cells (CD19^+^B220^+^AA4.1^−^CD1d^hi^IgM^hi^), GC B cells (CD19^+^B220^+^CD138^−^CD95^hi^CD38^lo/−^), plasma cells/plasmablasts (B220^−/lo^CD138^hi^), DZ GC B cells (CD19^+^B220^+^CD138^−^CD95^hi^CD38^lo/−^CXCR4^hi^CD86^lo^) and LZ GC B cells (CD19^+^B220^+^CD138^−^CD95^hi^CD38^lo/−^CXCR4^lo^CD86^hi^). For sorting B‐cell division cycles cells were harvested, filtered through 70 μm mesh filters (Corning, 352350) and resuspended in staining buffer.

Cell sorting was carried out on a FACS Aria III (BD Biosciences). Nonsinglet events were excluded from analyses based on characteristics of FSC‐H/FSC‐W and SSC‐H/SSC‐W.

### Quantitative real‐time‐PCR

RNA from snap‐frozen cell pellets of FACS‐sorted B cells was isolated using the Quick‐RNA Micro Prep Kit (Zymo Research, Irvine, CA, USA, R1050) and DNase digestion as per manufacturer's instructions. RNA from snap‐frozen *in vitro*‐cultivated B cells was isolated using Trizol reagent (Thermo Fisher Scientific, 15596026) as per manufacturer's instructions. DNA was removed using the RQ1 RNAse‐free DNase (Promega, Madison, WI, USA, M610A), and RNA was retrieved using GlycoBlue Coprecipitant (Thermo Fisher Scientific, AM9515) as per manufacturer's instructions. First‐strand cDNA was generated from 100 ng of total RNA using the iScript cDNA Synthesis Kit (Bio‐Rad, Hercules, CA, USA, 170‐8891) and cDNA was amplified using the AceQ qPCR SYBR Green Master Mix (Vazyme biotech, Nanjing, China, Q111‐02) as per manufacturer's instructions. The qRT‐PCR was run on a StepOnePlus Real‐time PCR system (Applied Biosystems, Foster City, CA, USA) and melt curve analysis was performed for every run. The expression of individual mRNAs was normalized to HPRT with the following formula: fold induction = $2^{\left(-\Delta C_{t}\right)}$, where $\Delta C_{t}=C_{t_{\left(\mathrm{target}\right)}}-C_{t_{\left(\mathrm{HPRT}\right)}}$. The following primers were used: HPRT F: 5′‐GTCATGCCGACCCGCAGTC‐3′, HPRT R: 5′‐AGTCCATGAGGAATAAAC‐3′; TET2 F: 5′‐GCCAGAAGCAAGAAACCAAG‐3′, TET2 R: 5′‐TTGGAGCAATGACAGTAGCC‐3′; TET3 F: 5′‐AAGAGTCTGCTGGACACACC‐3′, TET3 R: 5′‐CTCCATGAGTTCCCGGATAG‐3′; AID F: 5′‐GGACTTCGGCCACCTTC‐3′, AID R: 5′‐CATCTCAGAAACTCAGCCACG‐3′. Ki67 F: 5′‐GAACAGACTTGCTCTGGCCTAC‐3′, Ki67 R: 5′‐ CTTCATAGGCATTCCCTCACTC‐3′.

### ELISA

For NP‐specific serum IgG1 and IgM titres, ELISA was performed as described in 62. For total immunoglobulin levels in serum or secreted immunoglobulin in iGC culture medium, 50 μg·mL^−1^ capture antibody (Southern Biotech, 1010‐01) was coated overnight onto 96‐well enzyme‐linked immunosorbent assay plates at 4 °C (Sigma, CLS3590). Plates were washed three times with wash buffer (PBS containing 0.05% TWEEN 20), blocked with 100 μL per well 1% BSA in PBS for 4 h at room temperature and washed three more times with wash buffer. Subsequently, wells were incubated over night at 4 °C with 100 μL per well of mouse serum serially diluted 1 : 4 in blocking buffer (range 1 : 800 to 1 : 160 000). Plates were washed three times with wash buffer and incubated with 100 μL per well of HRP‐conjugated α‐mouse IgG1 (Southern Biotech, 1070‐05, 1 : 5000 in 1% BSA in PBS) or HRP‐conjugated α‐mouse IgM (Southern Biotech, 1020‐05, 1 : 5000 in 1% BSA in PBS) for 4 h at room temperature. For detection, 100 μL of ABTS substrate solution per well [200 μL ABTS (Stock: 15 mg·mL^−1^ in a.d.), 10 mL citrate‐phosphate buffer (574 mg citric acid monohydrate in 50 mL a.d.) and 10 μL H_2_O_2_] was incubated for 20 min. Absorbance was measured at 405 nm using a microplate reader (Tecan Sunrise, Männedorf, Switzerland).

### Immunohistochemistry

IHC was performed on 7 μm sections of murine spleens as described in Ref. 62. Imaging was performed on a Zeiss Axioplan 2 microscope using a 10× objective (Zeiss, Oberkochen, Gemany) and the Axiocam 305 colour camera (Zeiss). Pictures were generally processed with the zeiss zen blue 2.5 lite software (Zeiss).

### VH186.2 SHM analysis

Somatic hypermutation analysis was performed according to Ref. 63 for three Cg1‐Cre and three Cg1‐Cre*;Tet2* ^*F/F*^ *;Tet3* ^*F/F*^ mice. Briefly, IgG1^+^ GC B cells were FACS‐sorted (CD19^+^B220^+^CD138^−^Fas^hi^CD38^lo/−^IgG1^+^), pelleted and total RNA was isolated using the Quick‐RNA Micro Prep Kit (Zymo Research, R1050) including the DNase digestion step as per manufacturer's instructions. First‐strand cDNA was generated from 100 ng of total RNA using the Promega GoScript Reverse transcription system (Promega, A5000) and a gene‐specific primer for the IgG1 locus: Cg1 5′‐CATGGAGTTAGTTTGGGCAG‐3′. Subsequently, two rounds of semi‐nested PCRs were performed using the Herculase II Fusion DNA polymerase kit (Agilent, Santa Clara, CA, USA, 600675) as per manufacturer's instructions. Following each round of PCR, the gene product was run on an agarose gel and purified using the peqGold Gel extraction kit (VWR, Radnor, PA, USA, 732‐2777) as per manufacturer's instructions. For the first PCR, the following primers were used: V186.2 leader 5′‐AGCTGTATCATGCTCTTCTTGGCA‐3′, Cg1 5′‐CATGGAGTTAGTTTGGGCAG‐3′. For the second PCR, the following primers were used: V186.2 nested 5′‐CATGCTCTTCTTGGCAGCAACAG‐3′, Cg1 5′‐CATGGAGTTAGTTTGGGCAG‐3′. For cloning of VH186.2 segments, the pJet1.2 blunt vector was used and cleaved with EcoRV (New England BioLabs, Ipswich, MA, USA, R0195). The purified PCR products were ligated into the vector using T4 ligase (Promega, M1794) as per manufacturer's instructions. Using a standard transformation protocol, DH5α bacteria were transformed with the bulk of ligated plasmids, spread onto LB Agar/ampicillin plates and incubated over night at 37 °C. For each of the six mice, >10 colonies were transferred into a PCR Master Mix (Vazyme Biotech, P211), and the very same colonies were selected on new LB agar plates. For the colony PCR, the following primers were used: pJet1.2 F 5′‐CGACTCACTATAGGGAGAGCGGC‐3′, pJet1.2 R 5′‐AAGAACATCGATTTTCCATGGCAG‐3′. Cycle conditions were: 1 cycle: 95 °C for 1 min; 30 cycles: 95 °C for 15 s, 56 °C for 15 s, 72 °C for 45 s; 1 cycle: 72 °C for 3 min. The PCR products were loaded onto an agarose gel, and colonies with correct insertions were grown in 2 mL LB medium over night. Plasmid DNA was extracted using the Monarch Plasmid Miniprep Kit (New England BioLabs, T1010L) as per manufacturer's instructions. Subsequently, Sanger sequencing was performed using the pJet1.2 F primer detailed above. Sequences were aligned to the V_H_186.2 germ line sequence using VBASE2 (http://www.vbase2.org/). Mutation data were only used if the entire sequence was intact, and only unique sequences were included in the analysis. According to these criteria, two sequences from Cg1‐Cre*;Tet2* ^*F/F*^ *;Tet3* ^*F/F*^ GC B cells had to be excluded.

### Quantification and statistical analysis

Results are always shown as mean and standard deviation (SD). Graphs were plotted and statistical analysis was performed with graphpad prism 7 software (San Diego, CA, USA) using Student's *t* test when comparing two groups, One‐way ANOVA and Tukey *post hoc* test when comparing multiple groups and Mann–Whitney test for SHM analysis. The number of biological repetitions (*n*) is stated in each figure legend, and every experiment was performed at least twice. Differences between groups were considered statistically significant when *P* < 0.05. In figures, asterisks stand for: *P < 0.05; **P < 0.01; ***P < 0.001; ****P < 0.0001.

## Conflict of interest

The authors declare no conflict of interest.

## Author contributions

Conceptualization: VL; Methodology: VL; Investigation: KS, SM, SH, ED, AA; Resources: VL, SH, ED, AV, KR; Writing – Original Draft: VL; Visualization: VL; Project Administration: VL; Funding Acquisition: VL.
